# Some limit theorems for ratios of order statistics from uniform random variables

**DOI:** 10.1186/s13660-017-1569-7

**Published:** 2017-11-28

**Authors:** Shou-Fang Xu, Yu Miao

**Affiliations:** 1 Department of Statistics, School of Mathematics and Statistics, Xi’an Jiaotong University, Xi’an, P.R. China; 2 College of Mathematics and Information Science, Xinxiang University, Xinxiang, P.R. China; 3 College of Mathematics and Information Science, Henan Normal University, Xinxiang, P.R. China

**Keywords:** 60F15, 62G30, uniform distribution, order statistics, complete convergence, large deviation principle, almost sure central limit theorem

## Abstract

In this paper, we study the ratios of order statistics based on samples drawn from uniform distribution and establish some limit properties such as the almost sure central limit theorem, the large deviation principle, the Marcinkiewicz-Zygmund law of large numbers and complete convergence.

## Introduction

For a sample of independent observation $X_{1}, \ldots, X_{n}$ on a distribution *F*, the order sample values $X_{n(1)} \leq X_{n(2)} \leq \cdots \leq X_{n(n)}$ are called the order statistics. During the past many years, order statistics which is used to deal with properties and applications of ordered random variables and of functions of these variables appears in many statistical applications and are widely used in statistical modeling and inference based on some suitable functions of the order statistics. It might play a fundamental role in many diverse practical applications, for example, life-testing and reliability, robustness studies, statistical quality control, filtering theory, signal processing, image processing, radar target detection, and so on [1, 2].

The commonly used models of order statistics, such as the extreme values $X_{n(1)}$ and $X_{n(n)}$, the median, the sample range and the linear functions of order statistics are extensively studied and widely used in many fields. Recently, some scholars have paid increasing attention to the ratios of order statistics in theory, which can be used to measure the stability of areas of interest. For the convenience of description, let $\{X_{ni},1\leq i \leq m_{n},n\geq 1\}$ be an array of independent random variables drawn from distribution functions $F_{n}$. Denote by $\{X_{n(k)}, k=1, 2, \ldots, m_{n}\}$ the *k*th order statistics of the random variables $\{X_{ni},1\leq i \leq m_{n},n \geq 1\}$ for every given *n*, that is, $X_{n(1)} \leq X_{n(2)} \leq \cdots \leq X_{n(m_{n})}$. The scholars are interested in the following ratios of order statistics: $\{X_{n(k)},1\leq k \leq m_{n} \}$.

1.1 $$ R_{nij}=\frac{X_{n(j)}}{X_{n(i)}},\quad 1\leq i< j\leq m_{n}. $$

For some suitable probability distribution, there are a number of research results. We refer to Adler [3–5] for the order statistics from the Pareto distribution; refer to Miao *et al.* [6] and Zhang and Ding [7] for the order statistics from exponentials; refer to Adler [8] and Miao *et al.* [9] for the order statistics from the uniform distribution.

As is well known, the uniform distribution plays an important role in the theory of order statistics, because many problems about order statistics of samples from a general continuous distribution can be rephrased in terms of the set of order statistics from the uniform distribution by the technique which is called the quantile transformation. We refer to Chapter 6 of [10] for details. It is thus especially important to study uniform order statistics.

The aim of this paper is to further study the asymptotic properties of the ratios $R_{nij}$ of order statistics $\{X_{n(k)},1\leq k \leq m _{n}\}$ as the array of independent random variables $\{X_{ni},1 \leq i \leq m_{n},n\geq 1\}$ drawn from a sequence of uniform distributions $U(a_{n},b_{n})$ on the basis of the research by Adler [8] and Miao *et al.* [9]. Note that the ratio $R_{nij}$ takes value in $[1, \infty)$ and $[1, b_{n}/a _{n}]$ as $a_{n}=0$ and $a_{n} > 0$, respectively. Furthermore, as $a_{n} > 0$, the moments of the ratios $R_{nij}$ exist although they are cumbersome to use, so the asymptotic properties of the ratios $R_{nij}$ are ordinary in this case. Thus, we will focus on the asymptotic properties of the ratios $R_{nij}$ under $a_{n}=0$.

The layout of our work is as follows. Our main results are presented in Section 2 and the proofs of the main results are provided in Section 3. This paper is then ended with a brief summary. Throughout this paper, let $\log x =\ln \max \{e,x\}$ where ln is the natural logarithm, the symbol *C* denote a generic positive real number which is not necessarily the same in each appearance, and $I(A)$ represent the indicator function of the set *A*.

## Main results

The exact joint density function of the *i*th and *j*th order statistics of the random variables $\{X_{ni},1\leq i \leq m_{n},n\geq 1\}$ from $U(0, b_{n})$ for every given *n* is easily found.

$$f(x_{i},x_{j})=\frac{m_{n}! x_{i}^{i-1}(x_{j}-x_{i})^{j-i-1}(b_{n}-x _{j})^{m_{n}-j}}{(i-1)!(j-i-1)!(m_{n}-j)!b_{n}^{m_{n}}}I\quad (0\leq x_{i} < x _{j} \leq b_{n}). $$

Let $y=x_{i}$, $x=x_{j}/x_{i}$, then by the Jacobian we see that the joint density function of the *i*th order statistics $X_{n(i)}$ and the ratio $R_{nij}$ is

$$f(x,y)=\frac{m_{n}! y^{j-2}(x-1)^{j-i-1}(b_{n}-xy)^{m_{n}-j}}{(i-1)!(j-i-1)!(m _{n}-j)!b_{n}^{m_{n}}}I\quad (y\geq 0, x >1, xy \leq b_{n}). $$

Then it is easy to see that the density function of the ratio $R_{nij}$ is

2.1 $$ f_{R_{nij}}(x)=\frac{m_{n}!(x-1)^{j-i-1}}{(i-1)!(j-i-1)!(m_{n}-j)!b _{n}^{m_{n}}} \int_{0}^{b_{n}/x}y^{j-2}(b_{n}-xy)^{m_{n}-j} \,dy I\quad (x>1). $$

In this paper, we also study the three most representative ratios $R_{n12}$, $R_{n23}$ and $R_{n1j}$ based on the previous research.

### Asymptotic properties of $R_{n12}$

From (2.1), we can see that the density function of the ratio $R_{n12}$ is

$$f_{R_{n12}}(x)=\frac{1}{x^{2}} I\quad (x>1), $$

which is independent of *n*. Furthermore, we have $\mathbb{E} R_{n12} = \infty $ and $\mathbb{E} R_{n12}^{\beta } < \infty $ for all $\beta \in (0,1)$. It follows that the classical strong laws of $R_{n12}$ fail. Fortunately, Adler [8] and Miao *et al.* [9] studied the following analog of the ratio $R_{n12}$, which is called the exact law of large numbers or exact strong law.

If $0=a_{n} < b_{n}$, then, for all $\alpha > -2$,

$$\lim_{N \to \infty }\frac{1}{(\log N)^{\alpha + 2}}\sum_{n=1}^{N} \frac{( \log n)^{\alpha }}{n}R_{n12}=\frac{1}{\alpha +2}\quad \text{almost surely}. $$

Motivated by the above work, we naturally consider to establish the corresponding complete convergence of the ratio $R_{n12}$, which implies the above almost sure convergence by using the Borel-Cantelli lemma. Therefore, we first give the following complete convergence.

#### Theorem 2.1

*If* $0=a_{n} < b_{n}$ *and* $\alpha >-2$, *then*, *for any* $r>0$ *and every* $\delta > 0$,

$$\sum_{N=1}^{\infty }\frac{1}{N(\log N)^{\delta }}P \Biggl( \Biggl\vert \sum_{n=1}^{N} \frac{(\log n)^{\alpha }/n R_{n12}}{(\log N)^{\alpha + 2}}-\frac{1}{ \alpha +2} \Biggr\vert \geq r \Biggr) < \infty. $$

Note that

$$\lim_{x\to \infty }\exp \{\lambda x\} P ( R_{n12} > x) = \lim _{x\to \infty }\frac{\exp \{\lambda x\}}{x} = \infty $$

for all $\lambda > 0$, we say that the ratio $R_{n12}$ is heavy tailed. Large deviation techniques are very useful tools in different areas of probability theory, statistics, statistical physics, insurance mathematics, and other applied fields. For the heavy tailed random variables, the main stream of research has been centered on the study of the logarithmic asymptotic behaviors of the partial sums. Thus, we establish the following large deviation principle.

#### Theorem 2.2

*If* $0=a_{n} < b_{n}$, *then*, *for any* $x>1$,

$$\lim_{N \to \infty }\frac{1}{\log N}\log P \Biggl( \sum _{n=1}^{N} R_{n12} > N^{x} \Biggr) =-x+1. $$

### Asymptotic properties of $R_{n23}$

From (2.1), it is not difficult to check that the density function of the ratio $R_{n23}$ is

$$f_{R_{n23}}(x)=\frac{2}{x^{3}} I\quad (x>1), $$

which is independent of *n*. Unlike the ratio $R_{n12}$, the *β*-order moment of $R_{n23}$ exists for any $\beta \in (0,2)$ and the second moment is infinite. This enables us to study the classical strong laws of $R_{n23}$. Then we can give the following Marcinkiewicz-Zygmund law of large numbers.

#### Theorem 2.3

*If* $0= a_{n} < b_{n}$, *then*, *for any* $\delta \in (0,2)$, *we have*

$$\frac{\sum_{n=1}^{N}(R_{n23} - c)}{N^{1/\delta }} \to 0\quad \textit{a.s.} $$

*for some finite constant* *c*, *which takes value* 2 *as* $\delta \in [1,2)$ *while is arbitrary for* $\delta \in (0,1)$. *In particular*, *the classical strong law of large numbers holds as* $\delta =1$.

In previous research, Miao *et al.* [9] obtained the following central limit theorem of the ratio $R_{n23}$:

2.2 $$ \frac{1}{z_{N}} \sum_{n=1}^{N} ( R_{n23}-\mathbb{E} R_{n23} ) \xrightarrow{d} \Phi (x) \quad \text{as } N \to \infty, $$

where $\Phi (x)$ denotes the standard normal distribution, $z_{N} = 1 \lor \sup \{x >0; N L(x) \geq x^{2}\}$ and $L(x)=\mathbb{E} [ R ^{2}_{n23} I(\vert R_{n23}\vert \leq x) ] $ is a slowly varying function at ∞.

Berkes and Csáki [11] pointed out that not only the central limit theorem, but every weak limit theorem for independent random variables, subject to minor technical conditions, has an analogous almost sure version. This motivates us to study the almost sure central limit theorem of the ratio $R_{n12}$. Let us briefly recall the almost sure central limit theorem which is started by Brosamler [12] and Schatte [13]. In the past decade, there has been a lot of work on the almost sure central limit theorem and related ‘logarithmic’ limit theorems for partial sums of independent random variables. The simple form of the almost sure central limit theorem reads as follows. Let $\{X_{1}, X_{2}, \ldots \}$ be a sequence of i.i.d. random variables with mean 0 and variance 1 and denote $S_{n}=\sum_{i=1}^{n} X_{i}$, then

$$\lim_{N \to \infty }\frac{1}{\log N}\sum_{n=1}^{N} \frac{1}{n} I \biggl( \frac{S_{n}}{\sqrt{n}} \leq x \biggr) = \Phi (x)\quad \mbox{a.s.}, $$

for any real *x*. In this paper, we only need the simplest version under our model.

#### Theorem 2.4

*If* $0= a_{n} < b_{n}$, *then*, *for any real* *x*, *we have*

$$\lim_{N \to \infty }\frac{1}{\log N}\sum_{n=1}^{N} \frac{1}{n} I \biggl( \frac{S_{n}}{z_{n}} \leq x \biggr) = \Phi (x)\quad \textit{a.s.}, $$

*where* $S_{n}=\sum_{i=1}^{n} Y_{i} $, $Y_{n}= R_{n23}-\mathbb{E} R_{n23}$, $z_{n} = 1 \lor \sup \{x >0; n L(x) \geq x^{2}\}$ *and* $L(x)=\mathbb{E} (R^{2}_{n23} I(\vert R_{n23}\vert \leq x))$ *is a slowly varying function at* ∞.

It is easy to see that the ratio $R_{n23}$ is heavy tailed as well as $R_{n12}$. Thus, analogously to Theorem 2.2, we can also consider to establish the following large deviation principle of the ratio $R_{n23}$.

#### Theorem 2.5

*If* $0=a_{n} < b_{n}$, *then*, *for any* $x>1$,

$$\lim_{N \to \infty }\frac{1}{\log N}\log P \Biggl( \sum _{n=1}^{N} R_{n23} > N^{x} \Biggr) =-2x+1. $$

We would like to mention that the proof of Theorem 2.5 is very close to the proof of Theorem 2.2, so is omitted.

### Asymptotic properties of $R_{n1j}$

For the ratio of the first and *j*th order statistics when the sample size is fixed as *m*, it follows from (2.1) that the density function can be expressed as

$$f_{R_{n1j}}(x)=\frac{m!(x-1)^{j-2}}{(j-2)!(m-j)!x^{j}}\sum_{k=0}^{m-j} \left ( \begin{matrix} m-j \\ k \end{matrix} \right ) \frac{(-1)^{k}}{j+k}I\quad (x>1). $$

Obviously, it is dependent to *n* and the first moment of $R_{n1j}$ is infinite, in other words, the distribution of the ratio belongs to a Pareto family as well as $R_{n12}$. Thus, there are not classical strong laws of large numbers of $R_{n1j}$. Miao *et al.* [9] obtained the following exact strong law.

If $0=a_{n} < b_{n}$, then, for all $\alpha > -2$,

$$\lim_{N \to \infty }\frac{\sum_{n=1}^{N}(\log n)^{\alpha }/n R_{n1j}}{( \log N)^{\alpha + 2}}=\frac{m!}{(j-2)!(m-j)!(\alpha +2)} \sum _{k=0} ^{m-j} \left ( \begin{matrix} m-j \\ k \end{matrix} \right ) \frac{(-1)^{k}}{j+k} \quad \text{a.s.} $$

Based on the above result we can establish the following complete convergence of the ratio $R_{n1j}$.

#### Theorem 2.6

*If* $0=a_{n} < b_{n}$, $2 \leq j \leq m$ *and* $\alpha >-2$, *then*, *for any* $r>0$ *and every* $\delta > 0$ *we have*

$$\sum_{N=1}^{\infty }\frac{1}{N(\ln N)^{\delta }}P \Biggl( \Biggl\vert \sum_{n=1} ^{N} \frac{(\log n)^{\alpha }/n R_{n1j}}{(\ln N)^{\alpha + 2}}-\frac{m! \gamma_{mj}}{(j-2)!(m-j)!(\alpha +2)} \Biggr\vert \geq r \Biggr) < \infty, $$

*where*

$$\gamma_{mj}=\sum_{k=0}^{m-j} \left ( \begin{matrix} m-j \\ k \end{matrix} \right ) \frac{(-1)^{k}}{j+k}. $$

For any $x>1$, we have

$$\begin{aligned} P ( R_{n1j} > x) =&\frac{m!\gamma_{mj}}{(j-2)!(m-j)!} \int _{x}^{\infty } \frac{(t-1)^{j-2}}{t^{j}}\,dt \\ =&\frac{m!\gamma_{mj}}{(j-2)!(m-j)!} \int_{x}^{\infty } \sum_{i=0} ^{j-2} \left ( \begin{matrix} j-2 \\ i \end{matrix} \right ) (-1)^{i} t^{-i-2}\,dt \\ =& \frac{m!\gamma_{mj}}{(j-2)!(m-j)!} \sum_{i=0}^{j-2} \left ( \begin{matrix} j-2 \\ i \end{matrix} \right ) \frac{(-1)^{i}}{i+1} x^{-i-1} , \end{aligned}$$

which implies

$$\lim_{x\to \infty }\exp \{\lambda x\} P ( R_{n1j} > x) = \infty\quad \text{for all } \lambda > 0. $$

It follows that the ratio $R_{n1j}$ is heavy tailed too. Thus, we naturally consider to establish the following large deviation principle of the ratio $R_{n1j}$.

#### Theorem 2.7

*If* $0=a_{n} < b_{n}$, *then*, *for any* $x>1$,

$$\lim_{N \to \infty }\frac{1}{\log N}\log P \Biggl( \sum _{n=1}^{N} R_{n1j} > N^{x} \Biggr) =-x+1. $$

## Proofs of main results

To prove the complete convergence of $R_{n12}$ and $R_{n1j}$, the following lemma obtained by Sung *et al.* [14] will be used.

### Lemma 3.1

*Let* $\{X_{ni},1 \leq i \leq m_{n} ,n \geq 1\}$ *be an array of rowwise independent random variables and* $\{d_{n}, n \geq 1\}$ *a sequence of positive constants such that* $\sum_{n=1}^{\infty } d_{n} = \infty $. *Suppose that for every* $r >0$ *and some* $\varepsilon >0 $:

(i) $\sum_{n=1}^{\infty } d_{n} \sum_{i=1}^{m_{n}} P(\vert X_{ni}\vert > r) < \infty$,
(ii) *there exists* $J \geq 2 $ *such that*
  $$\sum_{n=1}^{\infty } d_{n} \Biggl( \sum_{i=1}^{m_{n}} \mathbb{E} X_{ni} ^{2} I \bigl(\vert X_{ni}\vert \leq \varepsilon \bigr) \Biggr) ^{J} < \infty, $$
(iii) $\sum_{i=1}^{m_{n}} \mathbb{E} X_{ni} I(\vert X_{ni}\vert \leq \varepsilon) \to 0$ *as* $n \to \infty $.

*Then we have*

$$\sum_{n=1}^{\infty } d_{n} P \Biggl( \Biggl\vert \sum_{i=1}^{m_{n}} X_{ni} \Biggr\vert > r \Biggr) < \infty\quad \textit{for all } r >0. $$

### Proof of Theorem 2.1

Let $c_{n} = n (\log n)^{2}$. It is easy to see that

3.1 $$ \sum_{n=1}^{N} \frac{(\log n)^{\alpha }/n R_{n12}}{(\log N)^{\alpha + 2}} =: I_{N} + II_{N} + III_{N}, $$

where

$$\begin{aligned}& I_{N} := \sum_{n=1}^{N} \frac{(\log n)^{\alpha }/n [R_{n12} I(1 \leq R_{n12} \leq c_{n} )- \mathbb{E} R_{n12} I(1 \leq R_{n12} \leq c _{n} ) ]}{(\log N)^{\alpha + 2}}, \\& II_{N} := \sum_{n=1}^{N} \frac{(\log n)^{\alpha }/n R_{n12} I(R_{n12} > c_{n} )}{(\log N)^{\alpha + 2}}, \\& III_{N} := \sum_{n=1}^{N} \frac{(\log n)^{\alpha }/n \mathbb{E} R _{n12} I(1 \leq R_{n12} \leq c_{n} ) }{(\log N)^{\alpha + 2}}. \end{aligned}$$

By simple calculation, we have

$$\mathbb{E} R_{n12} I(1 \leq R_{n12} \leq c_{n} )= \log c_{n} \thicksim \log n, $$

which implies $III_{N} \thicksim \frac{1}{\alpha + 2}$. Similarly, from the fact that

$$\mathbb{E} R_{n12}^{2} I(1 \leq R_{n12} \leq c_{n} )=n (\log n)^{2} -1 $$

and the Markov inequality, we have

$$P \bigl(\vert I_{N} \vert \geq r \bigr) \leq \frac{C}{\log N} \quad \text{for any } r >0. $$

Combining the conclusions of $I_{N}$ and $III_{N}$, we can see that

3.2 $$ \sum_{N=1}^{\infty } \frac{1}{N(\log N)^{\delta }}P \biggl( \biggl\vert I_{N} + III_{N} - \frac{1}{\alpha +2} \biggr\vert \geq r \biggr) < \infty. $$

To complete the proof of this theorem, it is sufficient to prove

3.3 $$ \sum_{N=1}^{\infty } \frac{1}{N(\log N)^{\delta }}P \bigl( \vert II_{N} \vert \geq r \bigr) < \infty. $$

By using Lemma 3.1 with

$$X_{Nn}=\frac{(\log n)^{\alpha }/n R_{n12} I(R_{n12} > c_{n} )}{( \log N)^{\alpha + 2}}\quad \text{and}\quad d_{N}= \frac{1}{N(\log N)^{\delta }}, $$

we only need to verify the following three conditions: (3.4)-(3.6). Firstly, for every $r>0$,

$$\begin{aligned} \sum_{n=1}^{N}P(X_{Nn} \geq r) \leq & \sum_{n=1}^{N}P \biggl( R_{n12} \geq \max \biggl\{ c_{n}, \frac{n r (\log N)^{\alpha +2} }{(\log n)^{ \alpha }} \biggr\} \biggr) \\ =& \sum_{n=1}^{N} \int_{\xi }^{\infty } \frac{1}{x^{2}}\,dx \\ \leq & C \sum_{n=1}^{N}\max \biggl\{ \frac{1}{n(\log n)^{2}}, \frac{( \log n)^{\alpha }}{n r (\log N)^{\alpha +2} } \biggr\} \leq \frac{C}{ \log N} , \end{aligned}$$

where $\xi =\max \{ c_{n}, n r (\log N)^{\alpha +2}/(\log n)^{ \alpha } \} $. It follows that

3.4 $$ \sum_{N=1}^{\infty } d_{N} \sum_{n=1}^{N}P(X_{Nn} \geq r) < \infty. $$

Secondly, we obtain

3.5 $$ \sum_{N=1}^{\infty } d_{N} \Biggl( \sum_{n=1}^{N} \mathbb{E} X_{Nn}^{2} I(X_{Nn} \leq \varepsilon) \Biggr) ^{2} < \infty, $$

where we used the fact that

$$\sum_{n=1}^{N} \mathbb{E} X_{Nn}^{2} I(X_{Nn} \leq \varepsilon) \leq \frac{C}{(\log N)^{\alpha +2}} \sum_{n=1}^{N} \frac{(\log n)^{ \alpha }}{n} \leq \frac{C}{\log N}. $$

Finally, for any $\varepsilon >0$, we have

$$\sum_{n=1}^{N} \mathbb{E} X_{Nn} I(X_{Nn} \leq \varepsilon)\leq C \frac{ \log \log N}{(\log N)^{\alpha +2}}\sum _{n=1}^{N} \frac{(\log n)^{ \alpha }}{n}\leq C \frac{\log \log N}{\log N}, $$

which implies

3.6 $$ \sum_{n=1}^{N} \mathbb{E} X_{Nn} I(X_{Nn} \leq \varepsilon) \to 0 \quad \text{as } N \to \infty. $$

The proof is completed. □

### Proof of Theorem 2.2

For any $x>1$, it is easy to see that

$$P \bigl( R_{n12} > N^{x} \bigr) =N^{-x}. $$

Thus, we have

$$\begin{aligned} P \Biggl( \sum_{n=1}^{N} R_{n12} > N^{x} \Biggr) \geq & P \bigl( \text{there exists at least a $n\in [1,N]$ such that } R_{n12} > N^{x} \bigr) \\ = & P \biggl( \bigcup_{1 \leq n \leq N} \bigl\{ R_{n12} > N^{x} \bigr\} \biggr) \\ = & \sum_{n=1}^{N} P \bigl( R_{n12} > N^{x} \bigr) = N^{-x+1}. \end{aligned}$$

It follows that

3.7 $$ \liminf_{N \to \infty }\frac{1}{\log N}\log P \Biggl( \sum_{n=1}^{N} R _{n12} > N^{x} \Biggr) \geq -x+1. $$

Next, we shall show that, for any $x>1$,

3.8 $$ \limsup_{N \to \infty }\frac{1}{\log N}\log P \Biggl( \sum_{n=1}^{N} R _{n12} > N^{x} \Biggr) \leq -x+1. $$

Denote $\tilde{R}_{n12}=R_{n12} I(R_{n12}\leq N^{x}/\upsilon^{1/ \beta })$, where $0< \beta <1$, $\upsilon >0$ and $x > 1$. Notice that

$$\Biggl\{ \sum_{n=1}^{N} R_{n12} > N^{x} \Biggr\} \subset \Bigl\{ \mathop{\bigcup } _{n=1}^{N} \bigl\{ R_{n12}> N^{x}/ \upsilon^{1/\beta } \bigr\} \Bigr\} \cup \Biggl\{ \sum _{n=1}^{N} \tilde{R}_{n12}> N^{x} \Biggr\} , $$

which implies

3.9 $$ P \Biggl( \sum_{n=1}^{N} R_{n12} > N^{x} \Biggr) \leq \sum _{n=1}^{N} P \bigl( R_{n12}> N^{x}/\upsilon^{1/\beta } \bigr) + P \Biggl( \sum _{n=1} ^{N} \tilde{R}_{n12}> N^{x} \Biggr). $$

Since $P ( R_{n12} > N^{x}/\upsilon^{1/\beta } ) = \upsilon^{1/\beta }N^{-x}$, we obtain for every *N*,

$$\log \Biggl[ \sum_{n=1}^{N} P \bigl( R_{n12} > N^{x}/\upsilon^{1/\beta } \bigr) \Biggr] = \beta^{-1}\log \upsilon +(-x+1) \log N. $$

It follows that the first term of the right-hand side in (3.9) satisfies

3.10 $$ \limsup_{N \to \infty }\frac{1}{\log N}\log \Biggl[ \sum_{n=1}^{N} P \bigl( R_{n12} > N^{x}/\upsilon^{1/\beta } \bigr) \Biggr] = -x+1. $$

Next, we are concerned with the second term of the right-hand side in (3.9). By simple calculation, we have, for every $1 \leq n \leq N $ and any $\lambda >0$,

$$\begin{aligned} \mathbb{E} \bigl( \exp \{\lambda \tilde{R}_{n12}\} \bigr) =& \mathbb{E} \biggl( \frac{\exp \{\lambda \tilde{R}_{n12}\}-1}{\tilde{R} _{n12}^{\beta }}\tilde{R}_{n12}^{\beta } \biggr) + 1 \\ \leq & \frac{\exp \{\lambda N^{x}/\upsilon^{1/\beta }\}-1}{N^{ \beta x}/\upsilon }\mathbb{E} \tilde{R}_{n12}^{\beta } + 1 \\ \leq & \exp \biggl\{ \frac{\exp \{\lambda N^{x}/\upsilon^{1/\beta }\}-1}{N ^{\beta x}/\upsilon }\mathbb{E} \tilde{R}_{n12}^{\beta } \biggr\} \\ \leq & \exp \biggl\{ \frac{\exp \{\lambda N^{x}/\upsilon^{1/\beta }\}-1}{N ^{\beta x}/\upsilon }\mathbb{E} R_{n12}^{\beta } \biggr\} . \end{aligned}$$

Then, by Markov’s inequality and the fact that $\mathbb{E} R_{n12} ^{\beta }=(1- \beta)^{-1}$, we have

$$\begin{aligned} P \Biggl( \sum_{n=1}^{N} \tilde{R}_{n12}> N^{x} \Biggr) \leq & \exp \bigl\{ - \lambda N^{x} \bigr\} \prod_{n=1}^{N} \mathbb{E} \bigl( \exp \{\lambda \tilde{R}_{n12}\} \bigr) \\ \leq & \exp \biggl\{ - \lambda N^{x} + \frac{\exp \{\lambda N^{x}/ \upsilon^{1/\beta }\}-1}{N^{\beta x}/\upsilon } N(1- \beta)^{-1} \biggr\} \\ \leq & \exp \bigl\{ \upsilon^{1/\beta } \bigr\} \bigl[ (1- \beta)^{-1}N ^{1-\beta x }\upsilon^{1-1/\beta } \bigr] ^{\upsilon^{1/\beta }} \end{aligned}$$

by taking $\lambda = \upsilon^{1/\beta }/N^{x} [(1/\beta -1)\log \upsilon + (\beta x -1)\log N + \log (1-\beta)]$. It follows that

3.11 $$ \limsup_{N \to \infty }\frac{1}{\log N}\log P \Biggl( \sum_{n=1}^{N} \tilde{R}_{n12}> N^{x} \Biggr) \leq \upsilon^{1/\beta }(1-\beta x). $$

From (3.9)-(3.11) and the elementary inequality

$$\log (a+b) \leq \log 2 +\max \{\log a,\log b\} $$

for all $a>0$ and $b>0$, we have

3.12 $$ \limsup_{N \to \infty }\frac{1}{\log N}\log P \Biggl( \sum_{n=1}^{N} R _{n12} \geq N^{x} \Biggr) \leq \max \bigl\{ -x+1,\upsilon^{1/\beta }(1- \beta x) \bigr\} . $$

At last, by choosing suitable *β* and *υ* such that $\upsilon^{1/\beta }(1-\beta x)\leq -x+1$, we obtain the desired result (3.8). The proof is completed. □

### Proof of Theorem 2.3

For any $\delta \in (0,2)$, we obtain

$$\mathbb{E} \vert R_{n23}\vert ^{\delta }= \int_{1}^{\infty } \frac{2}{x^{3- \delta }}\,dx= \frac{2}{2-\delta } < \infty. $$

So by the Marcinkiewicz-Zygmund theorem, which can be found on page 125 in [15], we have the desired result. □

### Proof of Theorem 2.4

Denote

$$Y_{Nn}'=Y_{n} I \bigl(\vert Y_{n} \vert >z_{N} \bigr)-\mathbb{E} \bigl[Y_{n} I \bigl( \vert Y_{n}\vert >z_{N} \bigr) \bigr] $$

and

$$Y_{Nn}''=Y_{n} I \bigl(\vert Y_{n}\vert \leq z_{N} \bigr)-\mathbb{E} \bigl[Y_{n} I \bigl(\vert Y_{n}\vert \leq z _{N} \bigr) \bigr]. $$

Obviously, we have

$$S_{N}=\sum_{n=1}^{N} Y_{Nn}'+ \sum_{n=1}^{N} Y_{Nn}''=:S_{N}' + S_{N}''. $$

Note that, for any $\varepsilon >0$ and all $x \in \mathbb{R}$,

$$\begin{aligned}& I \biggl( \frac{S_{n}''}{z_{n}} \leq x-\varepsilon \biggr) - I \biggl( \biggl\vert \frac{S_{n}'}{z_{n}} \biggr\vert > \varepsilon \biggr) \\& \quad \leq I \biggl( \biggl\{ \frac{S_{n}''}{z_{n}} \leq x-\varepsilon \biggr\} \cap \biggl\{ \biggl\vert \frac{S_{n}'}{z_{n}} \biggr\vert < \varepsilon \biggr\} \biggr) \\& \quad \leq I \biggl\{ \frac{S_{n}}{z_{n}} \leq x \biggr\} \leq I \biggl( \frac{S _{n}''}{z_{n}} \leq x + \varepsilon \biggr) + I \biggl( \biggl\vert \frac{S _{n}'}{z_{n}} \biggr\vert > \varepsilon \biggr). \end{aligned}$$

Therefore, to obtain the desired result, it is enough to show that

3.13 $$ \lim_{N \to \infty }\frac{1}{\log N}\sum _{n=1}^{N} \frac{1}{n} I \biggl( \frac{S_{n}''}{z_{n}} \leq x \pm \varepsilon \biggr) = \Phi (x \pm \varepsilon) \quad \mbox{a.s.} $$

and

3.14 $$ \lim_{N \to \infty }\frac{1}{\log N}\sum _{n=1}^{N} \frac{1}{n} I \biggl( \biggl\vert \frac{S_{n}'}{z_{n}} \biggr\vert \geq \varepsilon \biggr) = 0\quad \mbox{a.s.} $$

Firstly, we shall prove (3.13). Notice that, for any *N*,

$$\frac{\mathbb{E} \vert S_{N}'\vert }{z_{N}} \leq \frac{2 N }{z_{N}} \mathbb{E} \bigl[ \vert Y_{1}\vert I \bigl(\vert Y_{1}\vert >z_{N} \bigr) \bigr] \leq 0. $$

It follows from Markov’s inequality that $S_{N}'/z_{N} \to 0$ in probability as $N \to \infty $. Then by the Slutsky theorem and (2.2), we have

3.15 $$ \frac{S_{N}''}{z_{N}}\xrightarrow{d} \Phi (x) \quad \text{as } N \to \infty. $$

Let *g* be a bounded Lipschitz function. It follows from (3.15) that

3.16 $$ \mathbb{E} g \biggl( \frac{S_{N}''}{z_{N}} \biggr) \to \mathbb{E} g \bigl(N(0,1) \bigr)\quad \text{as } N \to \infty. $$

On the other hand, notice that (3.13) is equivalent to (*cf.* Theorem 7.1 of Billingsley [16] and Section 2 of Lacey and Philipp [17])

3.17 $$ \lim_{N \to \infty }\frac{1}{\log N}\sum _{n=1}^{N} \frac{1}{n} g \biggl( \frac{S_{n}''}{z_{n}} \biggr) = \mathbb{E} g \bigl(N(0,1) \bigr) \quad \mbox{a.s.} $$

From (3.16), to prove (3.17), it is sufficient to show that, as $N \to \infty $,

3.18 $$ \frac{1}{\log N}\sum_{n=1}^{N} \frac{1}{n} \biggl[ g \biggl( \frac{S_{n}''}{z _{n}} \biggr) -\mathbb{E} g \biggl( \frac{S_{n}''}{z_{n}} \biggr) \biggr] =: \frac{1}{\log N}\sum _{n=1}^{N} \frac{1}{n} T_{n} \to 0 \quad \text{a.s.} $$

It is easy to see that

3.19 $$ \mathbb{E} \Biggl( \frac{1}{\log N}\sum _{n=1}^{N} \frac{1}{n} T_{n} \Biggr) ^{2}= \frac{1}{\log^{2} N} \Biggl( \sum _{n=1}^{N} \frac{1}{n^{2}} \mathbb{E} T_{n}^{2} + 2\sum_{n=1}^{N-1} \sum_{m=n+1}^{N} \frac{1}{nm} \mathbb{E} T_{n} T_{m} \Biggr). $$

Because *g* is a bounded Lipschitz function, the first term of the right-hand side in (3.19) satisfies

3.20 $$ \frac{1}{\log^{2} N} \sum_{n=1}^{N} \frac{1}{n^{2}}\mathbb{E} T_{n} ^{2} \leq \frac{C}{\log^{2} N} \sum_{n=1}^{N} \frac{1}{n^{2}} \leq \frac{C}{ \log N}. $$

Moreover, for $1 \leq n < m \leq N$, we have

3.21 $$\begin{aligned} \vert \mathbb{E} T_{n} T_{m} \vert = & \biggl\vert \operatorname{Cov}\biggl[ g \biggl( \frac{S_{n}''}{z _{n}} \biggr),g \biggl( \frac{S_{m}''}{z_{m}} \biggr) \biggr] \biggr\vert \\ = & \biggl\vert \operatorname{Cov}\biggl[ g \biggl( \frac{S_{n}''}{z_{n}} \biggr),g \biggl( \frac{S _{m}''}{z_{m}} \biggr) - g \biggl( \frac{S_{m}''-\sum_{i=1}^{n}Y_{mi}''}{z _{m}} \biggr) \biggr] \biggr\vert \\ \leq & \frac{C}{z_{m}} \mathbb{E} \Biggl\vert \sum _{i=1}^{n}Y_{mi}'' \Biggr\vert \leq \frac{C}{z_{m}} \Biggl( \mathbb{E} \Biggl\vert \sum _{i=1}^{n}Y _{mi}'' \Biggr\vert ^{2} \Biggr) ^{1/2} \\ \leq & \frac{\sqrt{n} C}{z_{m}} \bigl\{ \mathbb{E} \bigl[ Y_{1}^{2}I \bigl(\vert Y _{1}\vert \leq z_{m} \bigr) \bigr] \bigr\} ^{1/2}\leq \frac{\sqrt{n} C}{z_{m}} \sqrt{L(z_{m})}. \end{aligned}$$

In addition, it is easy to check that, as $m \to \infty $,

3.22 $$ z_{m} \to \infty \quad \text{and}\quad z_{m}^{2} \thicksim mL(z_{m}). $$

It follows from (3.21) and (3.22) that the last term of the right-hand side in (3.19) is

3.23 $$\begin{aligned} \frac{1}{\log^{2} N} \sum_{n=1}^{N-1} \sum_{m=n+1}^{N} \frac{1}{nm} \mathbb{E} T_{n} T_{m} \leq & \frac{C}{\log^{2} N} \sum _{n=1}^{N-1} \sum _{m=n+1}^{N} \frac{1}{n^{1/2}m^{3/2}} \\ = & \frac{C}{\log^{2} N} \sum_{m=2}^{N} \sum_{n=1}^{m-1} \frac{1}{n ^{1/2}m^{3/2}} \leq \frac{C}{\log N}. \end{aligned}$$

Thus, by (3.19), (3.20) and (3.23), we have

3.24 $$ \mathbb{E} \Biggl( \frac{1}{\log N}\sum _{n=1}^{N} \frac{1}{n} T_{n} \Biggr) ^{2} \leq \frac{C}{\log N}. $$

Take $N_{n}=e^{n^{\delta }}$, where $\delta > 1$. It follows from the Borel-Cantelli lemma and (3.24) that

3.25 $$ \lim_{n \to \infty }\frac{1}{\log N_{n}}\sum _{n=1}^{N_{n}} \frac{1}{n} \biggl[ g \biggl( \frac{S_{n}''}{z_{n}} \biggr) -\mathbb{E} g \biggl( \frac{S_{n}''}{z_{n}} \biggr) \biggr] =0 \quad \text{a.s.} $$

Then, from *g* is a bounded function and the fact that

$$\frac{\log N_{n+1}}{\log N_{n}}=\frac{(n+1)^{\delta }}{n^{\delta }} \to 1 \quad \text{as } n \to \infty, $$

we have, for $N_{n} < N \leq N_{n+1}$,

3.26 $$\begin{aligned}& \Biggl\vert \frac{1}{\log N}\sum _{n=1}^{N} \frac{1}{n} \biggl[ g \biggl( \frac{S _{n}''}{z_{n}} \biggr) -\mathbb{E} g \biggl( \frac{S_{n}''}{z_{n}} \biggr) \biggr] \Biggr\vert \\& \quad \leq \frac{1}{\log N_{n}} \Biggl\vert \sum_{n=1}^{N_{n}} \frac{1}{n} T _{n} \Biggr\vert +\frac{1}{\log N_{n}} \Biggl\vert \sum_{n=N_{n}+1}^{N_{n+1}} \frac{1}{n} T_{n} \Biggr\vert \\& \quad \leq \Biggl\vert \frac{1}{\log N_{n}}\sum_{n=1}^{N_{n}} \frac{1}{n} \biggl[ g \biggl( \frac{S_{n}''}{z_{n}} \biggr) -\mathbb{E} g \biggl( \frac{S _{n}''}{z_{n}} \biggr) \biggr] \Biggr\vert + \frac{C}{\log N_{n}} \sum _{n=N_{n}+1}^{N_{n+1}} \frac{1}{n}. \end{aligned}$$

A combination of (3.25) with (3.26) yields the desired result (3.18). Therefore, the proof of (3.13) is completed.

Next, we show the proof of (3.14). Let *h* be a real-valued function satisfying

$$I(x \geq \varepsilon) \leq h(x) \leq I(x \geq \varepsilon /2) \sup _{x} \bigl\vert h'(x) \bigr\vert < \infty, $$

where $h'(x)$ is the first derivative of $h(x)$. Thus, we have

3.27 $$ I \biggl( \frac{S_{n}'}{z_{n}} > \varepsilon \biggr) \leq h \biggl( \frac{S _{n}'}{z_{n}} \biggr) \leq h \biggl( \frac{S_{n}'}{z_{n}} \biggr) - \mathbb{E} h \biggl( \frac{S_{n}'}{z_{n}} \biggr) + \mathbb{E} h \biggl( \frac{S _{n}'}{z_{n}} \biggr). $$

By the same argument used to obtain (3.17), we can see that (3.14) in the case $\{S_{n}'/z_{n} > \varepsilon \}$ is equivalent to

3.28 $$ \lim_{N \to \infty }\frac{1}{\log N}\sum _{n=1}^{N} \frac{1}{n} h \biggl( \frac{S_{n}'}{z_{n}} \biggr) = 0\quad \mbox{a.s.} $$

To prove (3.28), first we shall prove that as $N \to \infty $,

3.29 $$ \frac{1}{\log N}\sum_{n=1}^{N} \frac{1}{n} \biggl[ h \biggl( \frac{S_{n}'}{z _{n}} \biggr) -\mathbb{E} h \biggl( \frac{S_{n}'}{z_{n}} \biggr) \biggr] \to 0 \quad \text{a.s.} $$

We would like to mention that the proof of (3.29) is a little bit different from the proof of (3.18). For $1 \leq n < m \leq N$, we have

3.30 $$\begin{aligned}& \biggl\vert \operatorname{Cov}\biggl[ h \biggl( \frac{S_{n}'}{z_{n}} \biggr),h \biggl( \frac{S _{m}'}{z_{m}} \biggr) \biggr] \biggr\vert \\& \quad = \biggl\vert \operatorname{Cov}\biggl[ h \biggl( \frac{S _{n}'}{z_{n}} \biggr),h \biggl( \frac{S_{m}'}{z_{m}} \biggr) - h \biggl( \frac{S_{m}'-\sum_{i=1}^{n}Y_{mi}'}{z_{m}} \biggr) \biggr] \biggr\vert \\& \quad \leq \frac{C}{z_{m}} \mathbb{E} \Biggl\vert \sum _{i=1}^{n}Y_{mi}' \Biggr\vert \leq \frac{C n}{z_{m}} \mathbb{E} \bigl[ \vert Y_{1}\vert I \bigl(\vert Y_{1}\vert > z_{m} \bigr) \bigr] . \end{aligned}$$

Denote $\tilde{L}(x)=\mathbb{E} [ Y_{1}^{2} I(\vert Y_{1}\vert \leq x) ] $. Note that $\mathbb{E} Y_{1} =0$ and $\tilde{L}(x)$ is a slowly varying function at ∞, so by Lemma 1 of Csörgõ *et al.* [18], we have

3.31 $$ \mathbb{E} \bigl[ \vert Y_{1}\vert I \bigl(\vert Y_{1}\vert > z_{m} \bigr) \bigr] =o \biggl( \frac{1}{z _{m}}\tilde{L}(z_{m}) \biggr). $$

It follows from the facts that $\tilde{L}(x) \thicksim L(x)$ and $z_{m}^{2} \thicksim m L(z_{m})$ that

3.32 $$ \mathbb{E} \bigl[ \vert Y_{1}\vert I \bigl(\vert Y_{1}\vert > z_{m} \bigr) \bigr] =o \biggl( \frac{z_{m}}{m} \biggr). $$

From (3.30) and (3.32), we get

$$\frac{1}{\log^{2} N} \sum_{n=1}^{N-1}\sum _{m=n+1}^{N} \frac{1}{nm} \biggl\vert \operatorname{Cov}\biggl[ h \biggl( \frac{S_{n}'}{z_{n}} \biggr),h \biggl( \frac{S _{m}'}{z_{m}} \biggr) \biggr] \biggr\vert \leq \frac{C}{\log N}. $$

The rest of the proof for (3.29) is omitted. On the other hand, it follows from (3.31) that

$$P \biggl( \frac{S_{n}'}{z_{n}} \geq \frac{\varepsilon }{2} \biggr) \leq \frac{Cn}{ \varepsilon z_{n}} \mathbb{E} \bigl[ \vert Y_{1}\vert I \bigl( \vert Y_{1}\vert > z_{n} \bigr) \bigr] =o(1), $$

which implies

3.33 $$ \frac{1}{\log N}\sum_{n=1}^{N} \frac{1}{n} \mathbb{E} h \biggl( \frac{S _{n}'}{z_{n}} \biggr) \leq \frac{1}{\log N}\sum_{n=1}^{N} \frac{1}{n}P \biggl( \frac{S_{n}'}{z_{n}} \geq \frac{\varepsilon }{2} \biggr) \to 0\quad \text{as } N \to \infty. $$

From (3.27), (3.29) and (3.33), we have the desired result (3.28). Similarly, we can prove the desired result as $\{S_{n}'/z_{n} < - \varepsilon \}$. Thus, we obtain (3.14). The proof is completed. □

### Proof of Theorem 2.6

Let $c_{n} = n (\ln n)^{2}$. Note that

3.34 $$ \sum_{n=1}^{N} \frac{(\log n)^{\alpha }/n R_{n1j}}{(\ln N)^{\alpha + 2}} =: I_{N} + II _{N} + III_{N}, $$

where

$$\begin{aligned}& I_{N} := \sum_{n=1}^{N} \frac{(\log n)^{\alpha }/n [R_{n1j} I(1 \leq R_{n1j} \leq c_{n} )- \mathbb{E} R_{n1j} I(1 \leq R_{n1j} \leq c _{n} ) ]}{(\log N)^{\alpha + 2}}, \\& II_{N} := \sum_{n=1}^{N} \frac{(\log n)^{\alpha }/n R_{n1j} I(R_{n1j} > c_{n} )}{(\log N)^{\alpha + 2}}, \\& III_{N} := \sum_{n=1}^{N} \frac{(\log n)^{\alpha }/n \mathbb{E} R _{n1j} I(1 \leq R_{n1j} \leq c_{n} ) }{(\log N)^{\alpha + 2}}. \end{aligned}$$

For the first term in the partition (3.34), by combining Markov’s inequality and the fact that

$$\mathbb{E} R_{n1j}^{2} I(1 \leq R_{n1j} \leq c_{n} )=\frac{m!\gamma _{mj}}{(j-2)!(m-j)!} \int_{1}^{c_{n}}\frac{(x-1)^{j-2}}{x^{j-2}}\,dx \leq C \bigl(n( \log n)^{2} -1 \bigr), $$

we have, for any $r >0$,

3.35 $$ P \bigl(\vert I_{N} \vert \geq r \bigr) \leq C \sum_{n=1}^{N} \frac{ ( (\log n)^{\alpha }/n) ^{2}}{(\log N)^{2(\alpha +2)}} n(\log n)^{2} \leq \frac{C}{ \log N}. $$

For the third term in the partition (3.34), we have

$$\begin{aligned} \mathbb{E} R_{n1j} I(1 \leq R_{n12} \leq c_{n} ) =&\frac{m!\gamma_{mj}}{(j-2)!(m-j)!} \int_{1}^{c_{n}}\frac{(x-1)^{j-2}}{x ^{j-1}}\,dx \\ =& \frac{m!\gamma_{mj}}{(j-2)!(m-j)!}\sum_{i=0}^{j-2} \left ( \begin{matrix} j-2 \\ i \end{matrix} \right ) (-1)^{j-i-2} \int_{1}^{c_{n}} x^{i-j+1}\,dx \\ \thicksim& \frac{m!\gamma_{mj}}{(j-2)!(m-j)!} \log c_{n} \\ \thicksim& \frac{m!\gamma_{mj}}{(j-2)!(m-j)!} \log n, \end{aligned}$$

which implies

3.36 $$ III_{N} \thicksim \frac{m!\gamma_{mj}}{(j-2)!(m-j)!} \frac{1}{(\log N)^{ \alpha + 2}}\sum_{n=1}^{N} \frac{(\log n)^{\alpha +1}}{n} = \frac{m! \gamma_{mj}}{(j-2)!(m-j)!(\alpha +2)}. $$

From (3.35) and (3.36), we can see that

3.37 $$ \sum_{N=1}^{\infty } \frac{1}{N(\log N)^{\delta }}P \biggl( \biggl\vert I_{N} + III_{N} - \frac{m!\gamma_{mj}}{(j-2)!(m-j)!(\alpha +2)} \biggr\vert \geq r \biggr) < \infty. $$

At last, we consider the second term in the partition (3.34) by using Lemma 3.1 with

$$X_{Nn}=\frac{(\log n)^{\alpha }/n R_{n1j} I(R_{n1j} > c_{n} )}{( \log N)^{\alpha + 2}} \quad \text{and} \quad d_{N}= \frac{1}{N(\log N)^{\delta }}. $$

For any $r>0$, we have

$$\begin{aligned} \sum_{n=1}^{N}P(X_{Nn} \geq r) \leq & \sum_{n=1}^{N}P \biggl( R_{n1j} \geq \max \biggl\{ c_{n}, \frac{n r (\log N)^{\alpha +2} }{(\log n)^{ \alpha }} \biggr\} \biggr) \\ \leq & C \sum_{n=1}^{N} \int_{\xi }^{\infty } \frac{(x-1)^{j-2}}{x ^{j}}\,dx \\ \leq & C \sum_{n=1}^{N} \int_{\xi }^{\infty } \frac{1}{x^{2}}\,dx \\ \leq & C \sum_{n=1}^{N} \max \biggl\{ \frac{1}{c_{n}},\frac{(\log n)^{ \alpha }}{n r (\log N)^{\alpha +2} } \biggr\} \leq \frac{C}{\log N} , \end{aligned}$$

where $\xi =\max \{ c_{n}, n r (\log N)^{\alpha +2}/(\log n)^{ \alpha } \} $. It follows that

3.38 $$ \sum_{N=1}^{\infty } d_{N}\sum_{n=1}^{N}P(X_{Nn} \geq r) < \infty. $$

Similarly, for any $\varepsilon >0$, we see that

$$\begin{aligned} \sum_{n=1}^{N} \mathbb{E} X_{Nn}^{2} I(X_{Nn} \leq \varepsilon) \leq& \frac{C}{(\log N)^{\alpha +2}} \sum_{n=1}^{N} \frac{(\log n)^{ \alpha }}{n} \\ \leq& \frac{C}{\log N}, \end{aligned}$$

which implies

3.39 $$ \sum_{N=1}^{\infty } d_{N} \Biggl( \sum_{n=1}^{N} \mathbb{E} X_{Nn}^{2} I(X_{Nn} \leq \varepsilon) \Biggr) ^{2} < \infty. $$

In the same way, for any $\varepsilon >0$, we have

3.40 $$\begin{aligned} \sum_{n=1}^{N} \mathbb{E} X_{Nn} I(X_{Nn} \leq \varepsilon) \leq& C \frac{ \log \log N}{(\log N)^{\alpha +2}} \sum_{n=1}^{N} \frac{(\log n)^{ \alpha }}{n} \\ \leq& C \frac{\log \log N}{\log N} \to 0 \end{aligned}$$

as $N \to \infty $. Thus, from Lemma 3.1 and (3.38)-(3.40), we have

3.41 $$ \sum_{N=1}^{\infty } d_{N} P \bigl(\vert II_{N}\vert \geq r \bigr) < \infty. $$

A combination of (3.37) and (3.41) yields the desired result. The proof is completed. □

### Proof of Theorem 2.7

It is not difficult to see that, for any $x>0$,

$$\begin{aligned} P \bigl( R_{n1j} > N^{x} \bigr) =&\frac{m!\gamma_{mj}}{(j-2)!(m-j)!} \int_{N^{x}}^{\infty }\frac{(t-1)^{j-2}}{t^{j}}\,dt \\ =& \frac{m!\gamma_{mj}}{(j-2)!(m-j)!} \int_{N^{x}}^{\infty } t^{-2} \sum _{i=0}^{j-2} \left ( \begin{matrix} j-2 \\ i \end{matrix} \right ) (-1)^{i} t^{-i}\,dt \\ =& \frac{m!\gamma_{mj}}{(j-2)!(m-j)!} \left [ N^{-x} + \sum _{i=1}^{j-2} \left ( \begin{matrix} j-2 \\ i \end{matrix} \right ) \frac{(-1)^{i+1}}{i+1} N^{-(i+1)x} \right ] , \end{aligned}$$

which implies

3.42 $$ \lim_{N\to \infty }\frac{1}{\log N}\log P \bigl( R_{n1j} > N^{x} \bigr) =-x $$

and

3.43 $$ \lim_{N\to \infty }\frac{1}{\log N}\log \max _{1\leq n\leq N}P \bigl( R _{n1j} > N^{x} \bigr) =-x. $$

Let *r* be an arbitrary positive number. From (3.42), we have, for all *N* large enough,

$$P \bigl( R_{n1j} > N^{x} \bigr) \geq N^{-x-r}. $$

It follows that, for any $x>1$,

$$\begin{aligned} P \Biggl( \sum_{n=1}^{N} R_{n1j} > N^{x} \Biggr) \geq & P \bigl( \text{there exists at least a $n\in [1,N]$ such that } R_{n1j} > N^{x} \bigr) \\ = & P \biggl( \bigcup_{1 \leq n \leq N} \bigl\{ R_{n1j} > N^{x} \bigr\} \biggr) \\ = & \sum_{n=1}^{N} P \bigl( R_{n12} > N^{x} \bigr) \geq N^{-x-r+1}, \end{aligned}$$

which together with the arbitrariness of *r* yields

3.44 $$ \liminf_{N \to \infty }\frac{1}{\log N}\log P \Biggl( \sum_{n=1}^{N} R _{n1j} > N^{x} \Biggr) \geq -x+1. $$

Next we shall show that, for any $x>1$,

3.45 $$ \limsup_{N \to \infty }\frac{1}{\log N}\log P \Biggl( \sum_{n=1}^{N} R _{n1j} > N^{x} \Biggr) \leq -x+1. $$

Let $\tilde{R}_{n1j}=R_{n1j} I(R_{n1j}\leq N^{x}/\upsilon^{1/\beta })$, where $0< \beta <1$, $\upsilon >0$ and $x > 1$. Utilizing the approach in the proof of Theorem 2.2, we have

3.46 $$ P \Biggl( \sum_{n=1}^{N} R_{n1j} > N^{x} \Biggr) \leq \sum _{n=1}^{N} P \bigl( R_{n1j}> N^{x}/\upsilon^{1/\beta } \bigr) + P \Biggl( \sum _{n=1} ^{N} \tilde{R}_{n1j}> N^{x} \Biggr). $$

From (3.43), we have

$$\lim_{N\to \infty }\frac{1}{\log N}\log \max_{1\leq n\leq N}P \bigl( R _{n1j} > N^{x}/\upsilon^{1/\beta } \bigr) =-x. $$

It follows that the first term of the right-hand side in (3.46) satisfies

3.47 $$ \limsup_{N \to \infty }\frac{1}{\log N}\log \Biggl[ \sum_{n=1}^{N} P \bigl( R_{n1j} > N^{x}/\upsilon^{1/\beta } \bigr) \Biggr] \leq -x+1. $$

Next, we are concerned with the second term of the right-hand side in (3.46). It is easy to see that

$$\begin{aligned} \mathbb{E} R_{n1j}^{\beta } =&\frac{m!\gamma_{mj}}{(j-2)!(m-j)!} \int _{1}^{\infty } x^{\beta -2} \biggl( 1- \frac{1}{x} \biggr) ^{j-2}\,dx \\ =&\frac{m!\gamma_{mj}}{(j-2)!(m-j)!} \int_{1}^{\infty } \sum_{i=0} ^{j-2} \left ( \begin{matrix} j-2 \\ i \end{matrix} \right ) (-1)^{i} x^{-i+\beta -2}\,dt \\ =& \frac{m!\gamma_{mj}}{(j-2)!(m-j)!} \left [ \frac{1}{1-\beta } + \sum _{i=1}^{j-2} \left ( \begin{matrix} j-2 \\ i \end{matrix} \right ) \frac{(-1)^{i}}{i-\beta +1} \right ] , \end{aligned}$$

which implies

$$\sum_{n=1}^{N} \mathbb{E} R_{n1j}^{\beta } \leq CN. $$

Then, by simple calculation, we have, for any $\lambda >0$,

$$\begin{aligned} P \Biggl( \sum_{n=1}^{N} \tilde{R}_{n1j}> N^{x} \Biggr) \leq & \exp \bigl\{ - \lambda N^{x} \bigr\} \prod_{n=1}^{N} \mathbb{E} \bigl( \exp \{\lambda \tilde{R}_{n1j}\} \bigr) \\ \leq & \exp \Biggl\{ - \lambda N^{x} + \frac{\exp \{\lambda N^{x}/ \upsilon^{1/\beta }\}-1}{N^{\beta x}/\upsilon } \sum _{n=1}^{N} \mathbb{E} R_{n1j}^{\beta } \Biggr\} \\ \leq & C \exp \bigl\{ \upsilon^{1/\beta } \bigr\} \bigl[ N^{1-\beta x } \upsilon^{1-1/\beta } \bigr] ^{\upsilon^{1/\beta }} \end{aligned}$$

by taking $\lambda = \upsilon^{1/\beta }/N^{x} [(1/\beta -1)\log \upsilon + (\beta x -1)\log N + \log C]$. It follows that

3.48 $$ \limsup_{N \to \infty }\frac{1}{\log N}\log P \Biggl( \sum_{n=1}^{N} \tilde{R}_{n1j}> N^{x} \Biggr) \leq \upsilon^{1/\beta }(1-\beta x). $$

A combination of (3.46) with (3.47) and (3.48) yields

$$\limsup_{N \to \infty }\frac{1}{\log N}\log P \Biggl( \sum _{n=1}^{N} R _{n1j} \geq N^{x} \Biggr) \leq \max \bigl\{ -x+1,\upsilon^{1/\beta }(1- \beta x) \bigr\} . $$

This proves (3.45) by choosing suitable *β* and *υ* such that $\upsilon^{1/\beta }(1-\beta x)\leq -x+1$.

Finally, from (3.44) and (3.45), we can obtain the desired result. □

## Conclusion

There has been a growing interest in the ratios $R_{nij}$ of the order statistics from some suitable distribution. Because of the importance of the uniform distribution in order statistics, we investigate the ratios $R_{nij}$ of the order statistics from the uniform distribution in theory. As $a_{n}>0$, the moments of the ratios exist although they are cumbersome to use, thus the asymptotic properties of the ratios are ordinary in this case. So we focus on studying the ratios as $a_{n}=0$. Based on the research of predecessors, we established some asymptotic properties, which include the complete convergence and the large deviation principle of $R_{n12}$, the Marcinkiewicz-Zygmund law of large numbers, the almost sure central limit theorem and the large deviation principle of $R_{n23}$, the complete convergence and the large deviation principle of $R_{n1j}$. The results obtained in this paper might have a certain significance for advancing the study of the ratios of the order statistics.
